# Supplementary material for: Resolving the full spectrum of human genome variation using Linked-Reads
Source: Genome Res. 2019 Apr;29(4):635–45. doi: 10.1101/gr.234443.118 (PMC6442396; doi:10.1101/gr.234443.118)
Supplement: Supplemental Material [file supp_gr.234443.118_Supplemental_longranger-2.2.zip › longranger-2.2.tar.bz2!/lr2.2-public/lib/stan/cmdstan/stan/doc/api/html/math_2functions_2log__diff__exp_8hpp_source.html]

Stan: src/stan/math/functions/log\_diff\_exp.hpp Source File


|  |  |
| --- | --- |
|  | Stan  2.5.0  probability, sampling & optimization |


- Main Page
- Related Pages
- Namespaces
- Classes
- Files

- File List
- File Members

All Classes Namespaces Files Functions Variables Typedefs Enumerations Enumerator Friends Macros Pages

- src
- stan
- math
- functions

log\_diff\_exp.hpp

Go to the documentation of this file.

1 #ifndef STAN\_\_MATH\_\_FUNCTIONS\_\_LOG\_DIFF\_EXP\_HPP

2 #define STAN\_\_MATH\_\_FUNCTIONS\_\_LOG\_DIFF\_EXP\_HPP

3

4 #include <boost/math/tools/promotion.hpp>

5 #include <stdexcept>

6 #include <boost/throw\_exception.hpp>

7 #include <stan/math/functions/log1m\_exp.hpp>

8

9 namespace stan {

10  namespace math {

11

47  template <typename T1, typename T2>

48  inline typename boost::math::tools::promote\_args<T1,T2>::type

49  log\_diff\_exp(const T1 x, const T2 y) {

50  if (x <= y)

51  return std::numeric\_limits<double>::quiet\_NaN();

52  return x + log1m\_exp(y - x);

53  }

54

55  }

56 }

57

58 #endif

stan::math::log\_diff\_exp

boost::math::tools::promote\_args< T1, T2 >::type log\_diff\_exp(const T1 x, const T2 y)

The natural logarithm of the difference of the natural exponentiation of x1 and the natural exponenti...

**Definition:** log\_diff\_exp.hpp:49

log1m\_exp.hpp

stan::math::log1m\_exp

boost::math::tools::promote\_args< T >::type log1m\_exp(const T a)

Calculates the log of 1 minus the exponential of the specified value without overflow log1m\_exp(x) = ...

**Definition:** log1m\_exp.hpp:40


---

[ Stan Home Page ]

*© 2011–2014,
Stan Development Team.*
